# Molecular insights into the selective separation of perfluoroalkyl substances: The construction and application of capsule adsorbents

**DOI:** 10.1016/j.eehl.2026.100252

**Published:** 2026-06-10

**Authors:** Zhanghao Chen, Xinhao Wang, Junwen Qi, Liuqing Huang, Longgang Chu, Guixiang Zeng, Bing Wu, Juan Gao, Jiansheng Li, Cheng Gu, Hongqiang Ren

**Affiliations:** a State Key Laboratory of Pollution Control and Resource Reuse, School of Environment, Nanjing University, Nanjing, 210023, China; b Key Laboratory of Jiangsu Province for Chemical Pollution Control and Resources Reuse, School of Environmental and Biological Engineering, Nanjing University of Science and Technology, Nanjing, 210094, China; c Nanjing Institute of Environmental Sciences, Ministry of Environment and Ecology of China, Nanjing, 210042, China; d Kuang Yaming Honors School, Nanjing University, Nanjing, 210023, China; e Key Laboratory of Soil Environment and Pollution Remediation, Institute of Soil Science, Chinese Academy of Sciences, Nanjing, 210008, China

**Keywords:** PFAS, Porous material, Selective adsorption, Molecular mechanism, Fixed-bed tests, Water remediation

## Abstract

Perfluoroalkyl substances (PFAS) pollution has been a worldwide environmental challenge. Inspired by the semipermeable function of cell membranes, we constructed a millimeter-sized capsule adsorbent with specific molecular recognition capability to capture a broad spectrum of PFAS. This novel capsule consists of a core with a regulated nanoscale amine-rich network powder (ANP) and a porous polyvinylidene fluoride (PVDF) shell. Adsorption experiments and molecular simulations revealed that the PVDF shell acted as a semipermeable membrane, allowing only PFAS to pass through while repelling the coexisting environmental matrices, whereas the ANP core could provide the driving force for PFAS to enter the interior of the capsule adsorbent. Moreover, by regulating the hydrophilic functional structure of ANP, the driving force for short-chain PFAS to enter the capsule interior was enhanced, enabling the efficient capture of a wide range of PFAS (C−F number: 3−9). Further mechanistic studies demonstrated that the dual driving forces based on fluorophilicity and hydrogen bonding were key to capturing broad-spectrum PFAS. The fixed-bed experiments further demonstrated that the aminated organic fluorine capsule (AFC) column could treat approximately 18,000 bed volumes of effluent from synthetic PFOA-polluted water, with the effluent PFOA concentration remaining below 40 ng/L from an initial influent concentration of 1 μg/L (compared with the World Health Organization's drinking-water standard of 100 ng/L). Overall, this work not only provides a promising approach to treat PFAS-containing water but also sheds light on the design of functional remediation materials based on molecular properties and confined structures.

## Highlights

• The renewable porous aminated organic fluorine capsule was successfully prepared.
• AFC has semipermeable properties and can selectively enrich PFAS.
• Column experiments verified the applicability of AFC in PFAS removal.
• Broad-spectrum PFAS can be removed by regulating the molecular interactions.
• Molecular mechanism was proposed through experiments and computational simulations.

## Graphical abstract

**Figure:**
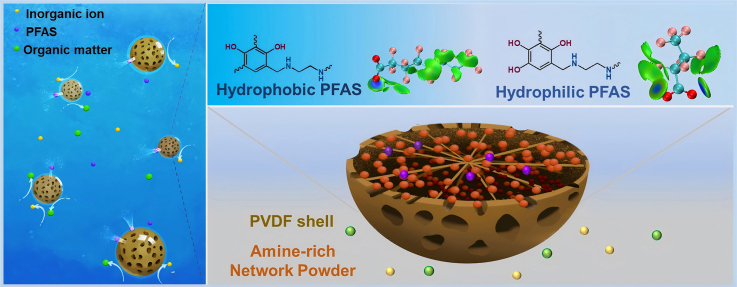

## 1 Introduction

Perfluoroalkyl substances (PFAS), including perfluoroalkyl carboxylic acids, perfluoroalkyl sulfonic acids, and perfluoroalkyl ether carboxylic acids, have been widely used to produce perfluoropolymers and textile coatings [1,2]. PFAS are also the main components of aqueous film-forming foams for firefighting [3]. Due to the resistance to biodegradation and advanced oxidation processes, PFAS are referred to as the forever chemicals [4,5]. Nowadays, PFAS, especially short-chain PFAS (chain length: 3 to 7), have been ubiquitously detected in natural and tap water from all over the world [[6], [7], [8]]. Long-term PFAS exposure (several ng/L to μg/L) has been linked to the occurrence of various adverse health outcomes, e.g., cancers [9], liver/renal damage [10], and endocrine disruption [11]. Typical PFAS, such as perfluorooctanoic acid (PFOA) and perfluorooctane sulfonate (PFOS), have been listed as persistent organic pollutants in the Stockholm Convention [12]. Moreover, the PFAS levels in drinking water have been regulated in several countries [[13], [14], [15]], with regulatory limits ranging from several to several tens of ng/L.

Although some degradation methods, e.g., hydrated electron-based advanced reduction process [16] and hydrothermal alkaline treatment [17], have been proven to effectively defluorinate PFAS, these systems are especially applicable for high-concentration PFAS wastewater without excessive impurities. Preconcentration is necessary for the treatment of trace PFAS pollution, and activated carbon [18], ion exchange resins (IX) [19], and reverse osmosis (RO) [20] have been identified as potential solutions to remediate impacted water by the U.S. Environmental Protection Agency (EPA). Based on these common commercial adsorbents, the molecular PFAS adsorption mechanism involving the synergistic control of hydrophobic and hydrophilic interactions was proposed, and the influence of different environmental factors on the different forces was discussed. However, due to their non-selectivity, carbon adsorbents are only effective against hydrophobic long-chain PFAS in pure water [21], while IX can easily lose its adsorption ability in the presence of other salts included in wastewater. Additionally, these technologies also suffer from the slow kinetics of adsorption, and longer water treatment time increases the capital and operational burden on water treatment facilities [22]. Although RO can remove most of the PFAS from water, it can produce a large volume of PFAS-containing brine and requires substantial energy input. Taken together, developing new adsorbents with high efficiency and broad-spectrum selectivity for PFAS and revealing the structure-property-performance mechanism are greatly needed for complex PFAS contaminations.

Selective adsorption is a critical challenge for water purification and environmental remediation [23], while the design of advanced functional materials is the key to addressing this issue [24,25]. Prevailing strategies for selective PFAS adsorption have focused on constructing fluorinated hydrophobic sites and aminated/hydrated hydrophilic sites, with a rough assumption that the fluorophilicity of organic fluorine moieties and the electron-withdrawing effects of hydrophilic sites are the key determinants of selectivity [26,27]. Based on this design principle, various functional adsorbents for PFAS have been synthesized, including fluoropolymer, fluorinated microgel, fluorinated covalent organic framework, and β-cyclodextrin [[28], [29], [30], [31]]. However, in our recent experiments, an unexpected phenomenon was observed when using polytetrafluoroethylene (PTFE) and/or polyvinylidene fluoride (PVDF) containers for PFAS studies: these materials, despite being rich in organic fluorine moieties, showed negligible PFAS adsorption, and a similar phenomenon was also observed in other laboratories [32,33]. This finding prompts a critical reconsideration: might there be potential undiscovered aspects in the molecular mechanism of selective PFAS adsorption that warrant further investigation? Apart from the unclear molecular mechanism, another practical challenge is the lack of PFAS-specific granular adsorbents with greater application potential, considering complicated synthesis procedures, high cost, and difficulty in recycling of the reported powdered adsorbents [[28], [29], [30]]. Therefore, developing adsorbents with practical potential and revealing the underlying molecular mechanism is greatly needed to address the emerging PFAS problems.

Herein, we proposed an adsorbent design strategy that combines the dual advantages of mimicking the function of a cell membrane, i.e., a fluorophilic selective barrier and a structurally tunable targeting core. Briefly, we leveraged the fluorophilicity of PFAS to construct a bionic semipermeable membrane and enhanced the driving force for capturing a broad-spectrum PFAS by regulating the functional structure of the internal core. PVDF, the fundamental building unit of common water treatment membrane [34] that contains abundant organic fluorine groups, was chosen to construct the selective barrier for PFAS. Moreover, amine-rich polymer network powder (ANP) was selected as the internal core, as its hydrophilicity could be easily regulated, and its amine functionality would provide high affinity for anionic PFAS [35,36]. In addition, competitive adsorption experiments, spectroscopic experiments, and molecular dynamics (MD) calculations have elucidated the roles of different groups in the selective adsorption of PFAS. Overall, this study not only elucidates the unidentified interaction mechanisms governing PFAS selective adsorption at the molecular scale but also provides a reliable solution to address the growing challenge of broad-spectrum PFAS contamination.

## 2 Materials and methods

The chemicals used in the experiments, detailed theoretical calculation methods, and adsorption procedures are described in Text S1.

### 2.1 Preparation of the ANP

ANP and polyhydroxy-ANP (H-ANP) were synthesized through a one-pot hydrothermal method [35]. An aqueous-alcoholic solution was prepared by mixing 16 mL of ethanol and 40 mL of distilled water. Subsequently, 0.6 mL of ethylenediamine was added under continuous stirring at room temperature. Then, 0.4 g of resorcinol/benzene-1,2,4-triol and 0.6 mL of 37% formaldehyde were added slowly, and the mixture was stirred for 24 h at 30 °C. The solid product was collected after centrifugation and then dried at 100 °C for 12 h. Finally, the obtained product was ground into powder (approximately 200 nm) for further use.

### 2.2 Preparation of AFCs and FC

Aminated organic fluorine capsules (AFCs) were synthesized through a solvent exchange process, as illustrated in Fig. S1 [37]. Briefly, 12 wt% PVDF, 2 wt% PVP, and different proportions of ANP were dissolved in DMF and stirred constantly at 60 °C for 8 h to obtain a uniform and homogeneous polymer solution. The proportions of PVDF and ANP were 12:2, 12:4, 12:6, 12:8, and 12:10, respectively. The remaining air bubbles that appeared due to the stirring of the casting solution were removed by ultrasonication. After that, the prepared homogeneous solution was injected dropwise into the coagulation bath, a mixture of deionized water and IPA, through a syringe needle using a syringe pump. Then, the freshly prepared AFCs were kept in pure water for more than 24 h to remove the residual solvent before being dried in an oven at 100 °C. The obtained AFCs with PVDF/ANP ratios of 12:2, 12:4, 12:6, 12:8, and 12:10 were named AFC-1, AFC-2, AFC-3, AFC-4, and AFC-5, respectively. The bulk fluorine capsule (FC) was synthesized following the same procedure without the addition of ANP. The optical images of bare organic FC and AFCs were collected as shown in Fig. S2.

### 2.3 Characterization and analysis

The surface morphologies of AFC and FC were characterized by scanning electron microscopy (SEM), and the SEM images were collected from a field-emission scanning electron microscope (FEI QUANTA FEG 250, USA). The surface areas and pore structures of AFC and FC were determined by N_2_ adsorption-desorption test based on the BJH model at 77 K (NOVA3000, Quantachrome, Boynton Beach, USA). XPS spectra were collected on an X-ray photoelectron spectrometer (PHI 5000 VersaProbe, Ulvac-PHI). Furthermore, fourier-transform infrared spectroscopy (FTIR) spectra were acquired with an FTIR spectrometer (Bruker Tensor 27, Germany). The procedure for measuring the concentrations of PFAS was described in our previous studies [38,39], and detailed characterization and analytical information are presented in Texts S2 and S3.

### 2.4 MD simulations

All-atom MD simulations were performed to characterize the detailed adsorption pathways of PFAS into AFC. Two fixed PVDF chains were used to simulate the PVDF membrane structure, and they were placed in the center of the box. On the left side of the PVDF chains, six monomolecular phenolic resins were placed to represent the inside of the resin spheres, and detailed information is presented in Text S4.

### 2.5 PFAS adsorption experiments

PFAS adsorption experiments, including kinetic and isotherm adsorption experiments, the effects of solution chemistry on the adsorption, the reusability of AFC, zeta potentials, and fixed-bed column experiments, are described in Texts S5-S7.

## 3 Results and discussion

### 3.1 Adsorbent characterization

**Fig. 1:**
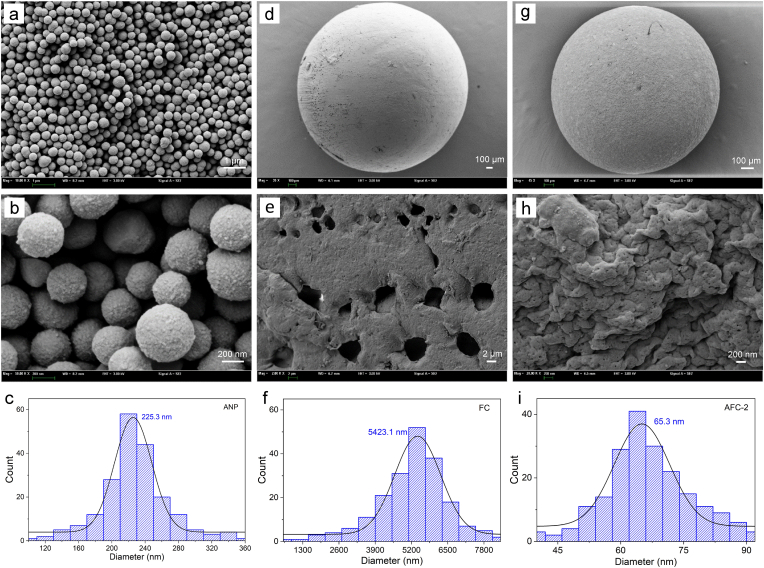
Representative morphology and surface structure of (a,b) ANP, (d,e) FC, and (g,h) AFC-2; the size distribution histograms of (c) ANP, (f) FC, and (i) AFC-2. ANP, amine-rich network powder; FC, fluorine capsule; AFC-2, aminated organic fluorine capsule with PVDF/ANP ratios of 12:4.

The surface morphologies and porous structures of bulk ANP, FC, and AFCs encapsulated with various amounts of ANP were first determined. As shown in Fig. 1a–c, the bulk ANP microspheres were uniform, with an average diameter of 225.3 ± 12.5 nm, while the diameter of bare FC is about 1.5–2 mm with abundant surface channels ranging from 1.2 to 8 μm with a median of 5.4 μm. After encapsulation of ANP, the resulting yellow-brown AFC particles were 1.5–1.8 mm in diameter, with a relatively uniform pore size of 28–330 nm (AFC-1 to AFC-4) by a statistical analysis on approximately 200 pores randomly selected from the SEM image (Figs. 1g–i and S3a-i). Moreover, the surface pores of AFC became smaller with the increase of ANP amount, which could be attributed to the blockage of pore channels by ANP [40]. Furthermore, for AFC-5, which had the highest amount of ANP, the surface pores could not be observed by SEM (Fig. S4a–b). The N_2_ adsorption-desorption tests for all samples were also conducted. As shown in Fig. S5a–h, for bulk FC, the Brunauer–Emmett–Teller (BET) surface area was 98.9 m^2^/g. After encapsulation of ANP, the surface areas of AFCs first increased and then decreased as more ANP was incorporated, and the largest BET surface area of 160.5 m^2^/g was observed for AFC-2. The increased BET surface areas of AFC-1 and AFC-2 could be explained by the more uniform micropores induced by the encapsulation of ANP [41]. However, a higher amount of ANP would obstruct the pore channel, thus reducing the specific surface area, as also supported by the gradual narrowing of the surface channels (Figs. 1 and S3). Therefore, it was expected that the larger BET surface area and more uniform surface pores of AFC-2 would increase the adsorption sites for pollutants.

Furthermore, the amine functional groups in both ANP and AFCs were characterized by spectroscopic analysis. The XPS spectra indicated the incorporation of ANP into the AFCs, as shown in Fig. S6. The XPS spectra of AFCs showed the characteristic amine N 1s peak at 400.1 eV [42], and the enhanced signal strength was observed with increased ANP loading. Moreover, high-resolution XPS spectra of ANP revealed that amine and imine were the main forms of N (Fig. S7). Furthermore, the encapsulation of ANP within the AFC was directly supported by cross-section SEM of AFC-2, as presented in Fig. S8. ANP was evenly dispersed in the AFC cavity. FTIR spectra also revealed that there were sufficient hydrocarbon groups on ANP, as indicated by C−H stretching at 2853.6 and 2923.9 cm^−1^ (Fig. S9) [43]. Compared to the bulk FC sample, the emergence of C−H stretching signals in AFCs demonstrated that the ANP was successfully encapsulated in AFCs. Collectively, our results demonstrate the formation of a capsule structure with ANP coated by a porous organic fluorine cover. The zeta potentials of bulk FC and ANP were also measured to assess their surface properties (Fig. S10). Importantly, the ANP was positively charged at pH values below 9.3, indicating that over a wide pH range, the positive charge would facilitate the long-distance migration of anionic PFAS into the AFC interior. In contrast, the isoelectric point of FC was 6.9.

### 3.2 PFAS removal and molecular interaction

The PFAS removal efficiency of each composite was evaluated by a batch adsorption experiment using PFOA as the model compound, with an initial PFOA ([PFOA]_0_) concentration of 1 μg/L and an adsorbent dosage of 1 g/L. As expected, each AFC exhibited superior removal ability for PFOA (Fig. S11). After 30 min of contact, all five AFCs could remove more than 80% of PFOA, while negligible PFOA removal was observed in the presence of bulk FC. The PFOA removal efficiency was strongly dependent on the ANP loadings in AFCs. For AFC-1 and AFC-2, the adsorption capacity was positively correlated with the amounts of ANP, suggesting that the dispersion of ANP in AFC was essential for surface affinity toward PFOA. However, with a further increase in the ANP loading, the adsorption decreased unexpectedly, which could be explained by the decrease in adsorption sites on AFCs as more ANP blocked the surface pores. During the adsorption process, *in situ* ATR-FTIR spectra of FC and ANP were collected at certain time intervals (Fig. S12). For ANP, the absorption peak at 1643.1 cm^−1^, corresponding to the vibration of the C=O bond of PFOA [44], increased continuously in the first 15 min and then remained constant, suggesting that the adsorption of PFOA onto ANP reached equilibrium within 15 min. However, after replacing ANP with bulk FC, only the baseline signal could be observed during the first 45 min, and a weak signal appeared at 60 min, indicating that FC had a much weaker affinity for PFOA than ANP (Fig. S12). In other words, ANP was essential for the rapid adsorption of PFOA by AFC. Therefore, the FTIR results also emphasized the importance of ANP to adsorb PFOA.

**Fig. 2:**
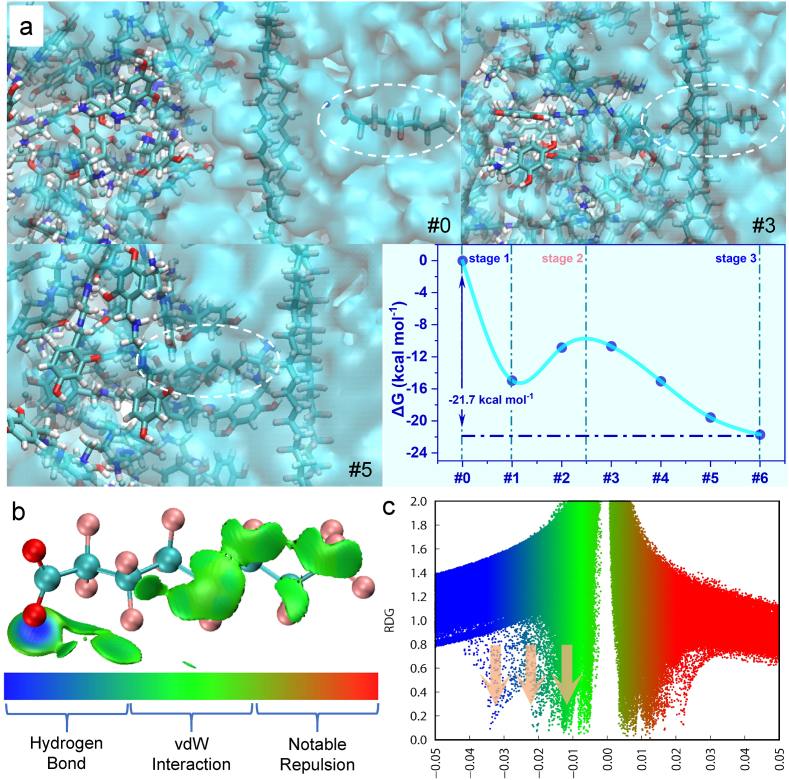
(A) Adsorption free-energy profiles computed for the PFOA penetration process from the bulk solution to the inner core of AFC; (b,c) average noncovalent interaction analysis of the interactions between PFOA and ANP.

To provide more intuitive insights into the significantly enhanced PFOA adsorption by AFCs, we investigated the adsorption process of PFOA onto AFC via all-atom MD simulation and calculated the binding free energies. The PFOA adsorption on AFC could be divided into three stages (Fig. 2a). In stage 1, the binding free energy dropped rapidly from 0 to −14.9 kcal/mol, indicating that the fluorophilic nature of PVDF played a major role in attracting PFOA to the AFC periphery. However, a distinct difference was observed in stage 2. As PFOA further penetrated the PVDF molecular layer, the binding free energy slightly increased. In this process, although PVDF continued to attract PFOA, the hydrophilic head of PFOA exhibited resistance to the hydrophobic PVDF, resulting in a penetration energy barrier (4.29 kcal/mol). However, the energy state remained significantly lower than that in bulk water. In other words, this process could occur spontaneously, especially when a large amount of PFOA was attracted to the PVDF surface, and the mechanical extrusion of PFOA molecules could accelerate the process. Moreover, a significant decrease in energy state (−21.7 kcal/mol) was observed when PFOA penetrated the PVDF layer and bound to the internal ANP core (stage 3). Collectively, given the overall change in the free energy throughout the adsorption process, the mass transfer of PFOA into AFC had a thermodynamic advantage. The decline in mass transfer energy state also indicated that the ANP internal core introduces a driving mode for PFOA adsorption. The exothermic adsorption process was further directly demonstrated by the temperature-effect experiments. As shown in Fig. S13, the amount of PFOA adsorption slightly decreased as the temperature increased. Therefore, we further amplified the molecular interaction between ANP and PFOA by optimizing their molecular binding configuration. As shown in Fig. 2b–c, the average noncovalent interaction analysis confirmed that both hydroxyl and amine functional groups on the ANP surface would interact simultaneously with the hydrophilic head and hydrophobic tail of PFOA with stable hydrogen bonding (blue isosurface and spike) and fluctuating van der Waals forces (green isosurface and spike), respectively, thereby directly generating the driving forces. Thus, the adsorbed PFOA would be locked by a double-binding morphology with the lowest energy state of −21.7 kcal/mol.

To gain further insights into the removal mechanism of PFOA by AFC, we constructed the adsorption isotherms of PFOA by AFCs with different amounts of ANP. All the isotherms were well-described by the Langmuir isotherm model (Fig. S14). Consistent with the PFOA removal ratios (Fig. S11), the maximum PFOA adsorption capacity was obtained for AFC-2 (Fig. S14). However, AFC-4 and AFC-5 exhibited a higher affinity coefficient (*K*_L_ = 0.10 and 0.096 L/mg) (Table S1). *K*_L_ can be used to describe the binding affinity between adsorbents and pollutants [13]. Since AFC-5 had the highest ANP loading, the crowded ANP sites could provide multiple interactions with PFOA [13], as demonstrated by the average noncovalent interactions analysis (Fig. S15). However, the loading amounts of ANP not only affected the adsorption sites on AFCs but also changed their surface pore structures. For AFC-4 and AFC-5, most ANPs were inactivated due to surface channel obstruction, resulting in reduced adsorption capacity for PFOA. Therefore, the highest PFOA adsorption capacity of AFC-2 could benefit from the optimal balance between the two aspects. We also benchmarked the PFOA adsorption by AFC-2 against the commercial granular activated carbon (GAC) at 1 g/L. As shown in Figs. S16 and S17, AFC-2 exhibited superior PFOA removal efficiency (approximately 97%, 5 h) with an equilibrium concentration of 20 ng/L, which was below the standard for drinking water in China and outperformed GAC (approximately 48%, 5 h). The adsorption capacity of AFC-2 was more than two times that of GAC (Table S1). Moreover, a significantly higher *K*_L_ value further proved that AFC-2 had a greater kinetic advantage than GAC for PFOA adsorption. Within 36 min, the PFOA concentration could be reduced from the initial 1 μg/L to about 50 ng/L by AFC-2. These observations indicate that AFC-2 is an outstanding candidate for PFOA treatment, while GAC is inefficient.

### 3.3 Selective adsorption and mechanism

**Fig. 3:**
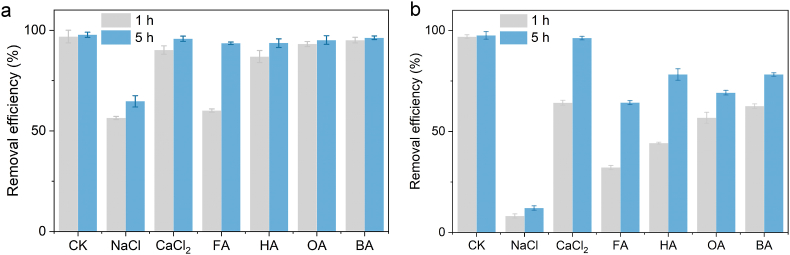
Competitive adsorption of PFOA by (a) AFC-2 and (b) ANP in the presence of various competitors. The initial concentration of PFOA was 1 μg/L, and the dosage of AFC-2 and ANP was 1 g/L; the concentrations of NaCl and CaCl_2_ were 10 mM, and the concentrations of FA, HA, OA, and BA were 5 mg/L. The temperature was controlled at 25 ± 1 °C on a rotary shaker at 150 rpm, and the solution pH was adjusted to 6.0 ± 0.1. FA, fulvic acid; HA, humic acid; OA, oxalic acid; BA, benzoic acid.

Natural water bodies or drinking water sources are generally complex in composition and usually contain relatively high concentrations of organic and/or inorganic coexisting substances [34]. Therefore, the adsorption selectivity of the adsorbent for PFAS removal is the key to evaluating its applicability. Herein, six common substances in the environmental water bodies, including oxalic acid (OA), benzoic acid (BA), natural organic matter [NOM; i.e., fulvic acid (FA) and humic acid (HA)], NaCl, and CaCl_2_, were selected to investigate their competitive effects on the adsorption of PFOA by AFC-2. As displayed in Fig. 3, the presence of coexisting 5 mg/L OA, BA, FA, or HA did not reduce the adsorption efficiency of AFC-2 for PFOA, although there were lots of nucleophilic carboxyl groups in OA, BA, FA, and HA. In contrast, the presence of 10 mM NaCl slightly inhibited the adsorption process (Fig. 3), probably due to the competition effect from Cl^−^. A similar phenomenon was also observed when using powdered activated carbon and surface-modified organoclay as adsorbents, and this was attributed to the interference with the hydrophilic interaction between PFAS and the adsorbent [45]. Surprisingly, the addition of CaCl_2_ did not affect PFOA removal by AFC-2, which could be attributed to the formation of PFOA-Ca complexes [46]. In contrast, the adsorption of PFOA on ANP was significantly inhibited in the presence of coexisting substances, except for CaCl_2_, demonstrating the important role of the PVDF porous shell in selective adsorption.

To intuitively compare the competitive adsorption of different substances on the AFCs, we also calculated the binding free energies of OA, BA, and Cl^−^ at key positions along their transport pathway from the bulk water phase to the AFC internal core through all-atom MD simulations (Figs. S18–S20). Different from PFOA, the penetration process of OA and BA into the AFC could be divided into two stages. In stage 1, the free energies of OA and BA increased sharply as they moved from the bulk solution to the PVDF surface due to the nonpolar repulsive force of the PVDF molecule, with the energy barriers of 24.1 and 28.5 kcal/mol, respectively, indicating that this process was endothermic and could not occur spontaneously. In stage 2, the penetration from the PVDF surface to the internal core was a spontaneous process, accompanied by the energy release of 41.2 and 62.4 kcal/mol, respectively. From a thermodynamic point of view, in the adsorption process, the PVDF network constructed a huge energy barrier for BA and OA. However, the fluorophilic nature of PVDF enabled spontaneous penetration of PFOA. Therefore, the PVDF layer served as a selective barrier for ANP, allowing only PFOA to pass. For Cl^−^, the penetration process was similar to that of BA and OA, but the penetration energy barrier was significantly lower (5.2 kcal/mol) due to its much smaller ionic radius, which could explain the slight inhibition of PFOA adsorption in the presence of NaCl.

Based on the above results and discussion, the selective PFAS adsorption mechanism of the AFC could be summarized as follows: for PFAS-contaminated water with high concentrations of low-molecular-weight organic acids and NOM, only PFOA can efficiently pass through the porous PVDF shell, which consists of a polymer with multiple C−F chains. In contrast, other organic substances are excluded from the shell. For different types of coexisting salts, different mechanisms may control the adsorption process of PFOA by AFC. In the presence of a high concentration of NaCl, Cl^−^ could compete with PFOA for adsorption sites (Fig. S21). Although Cl^−^ slightly suppressed the PFOA adsorption on AFC, the inhibitory effect was significantly weaker than that of bulk ANP (Fig. 3), demonstrating the buffering effect of the porous PVDF shell against Cl^−^ interference. Different from NaCl, the addition of Ca^2+^ can form a complex with the anionic PFOA, increasing the hydrophobicity of PFOA and subsequently enhancing the adsorption on AFC through hydrophobic interactions [47]. Therefore, the adsorption performance of AFC for PFOA was not significantly affected by the presence of CaCl_2_, even at a concentration up to 10 mM (Fig. 3). The selective adsorption mechanism of PFOA on AFC was also confirmed by *in situ* ATR-FTIR spectroscopy, and the detailed discussion is presented in Text S3, Figs. S22 and S23.

To further directly verify the anti-interference effect of porous PVDF shell on PFOA adsorption, AFCs with different pore sizes (from AFC-1 to AFC-5) were selected to adsorb PFOA in the presence of various environmental interferents (OA, BA, HA, FA, NaCl, and CaCl_2_). The pore size of the PVDF porous shell significantly affected the anti-interference performance of AFCs (Fig. S24). As expected, from AFC-1 to AFC-4, the resistance to coexisting substances during PFOA adsorption increased gradually with the decrease of surface pore size, demonstrating the significance of confined space for improving the adsorption selectivity of AFC. By contrast, for AFC-5, due to the blocking of the surface pores, the adsorption of PFOA mainly occurred on the surface. Therefore, this adsorption process was more easily disturbed by the coexisting substances in solution.

### 3.4 Broad-spectrum PFAS adsorption and pH influence

**Fig. 4:**
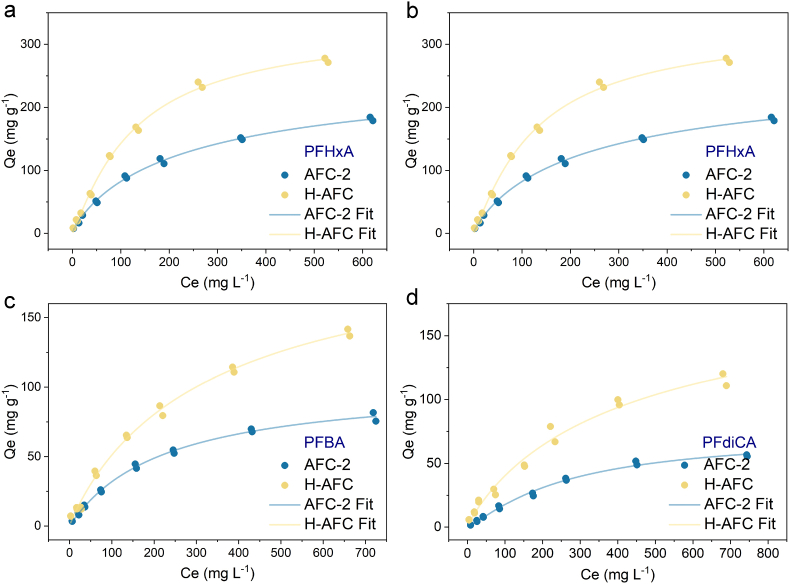
The Langmuir adsorption isotherms for (a) PFHxA, (b) PFBS, (c) PFBA, and (d) PFdiCA adsorbed by AFC-2 and H-AFC. The initial concentrations of these PFAS ranged from 10 to 800 mg/L. The adsorption experiment was conducted at room temperature (25 ± 1 °C) on a rotary shaker at 150 rpm for 12 h, and the solution pH was adjusted to 6.0 ± 0.1. H-AFC, polyhydroxy-aminated organic fluorine capsule.

**Fig. 5:**
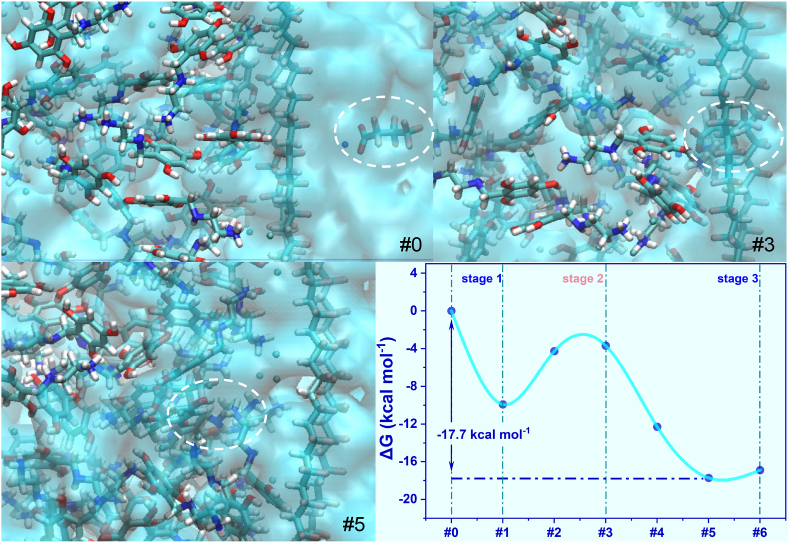
Adsorption free-energy profiles computed for the PFBA penetration process from the bulk solution to the H-AFC inner core.

Furthermore, the applicability of AFC-2 to the adsorption of broad-spectrum PFAS with different chain lengths and anionic functional groups at environmentally relevant concentrations was also investigated (Fig. S25). Under our experimental conditions, 4 of the 8 PFAS—PFOA, PFOS, hexafluoropropylene oxide trimer acid (HFPO-TA), and fluorotelomer carboxylic acid (FTCA)—could be removed at efficiencies >95% within 1 h, whereas approximately 70% removal was achieved for perfluorohexanoic acid (PFHxA), perfluorobutyric acid (PFBA), and perfluorobutane sulfonic acid (PFBS) (Fig. S25). However, for perfluorooctanedioic acid (PFdiCA) with double carboxyl groups, only about 40% removal efficiency was obtained (Fig. S25). Due to the strong polarity and hydrophilicity, these PFAS bind to AFC-2 with lower affinity through hydrophobic interactions [48], so their removal relies more on the hydrophilic interactions with the hydroxyl and amine functional groups on ANP. Based on the above exploration and findings, we therefore hypothesized that increasing the hydrogen-bond binding sites of ANP could enhance the capture efficiency of hydrophilic PFAS. We then synthesized H-ANP using benzene-1,2,4-triol (Fig. S26), and prepared the corresponding polyhydroxy-AFC (H-AFC) to evaluate its capture efficiency for a broad spectrum of PFAS. The adsorption tests for four different PFAS (PFHxA, PFBS, PFBA, and PFdiCA) were conducted, as presented in. Figs. 4 and S27. Notably, the maximum uptake capacities and removal efficiencies within 1 h increased from 73.7 to 265.7 mg/g and 41.2%−81.2% to 199.3−333.0 mg/g and 82.6%−96.2%, respectively, and the maximum adsorption capacities were 3−28 times higher than those of reported adsorbents [49,50]. Interestingly, the enhancement effect was more obvious for hydrophilic PFAS with lower log *K*_OW_ (Table S2). We further simulated the PFBA penetration process using ANP or H-ANP as the core of AFC (Figs. 5 and S28). The more hydrophilic H-ANP core reduced the adsorption free energy more significantly, which could be attributed to the introduction of more hydroxyl groups to strengthen hydrogen bonding with PFBA. Our results demonstrate that regulating the functional structure of ANP is an effective strategy to extend the adsorption ability of AFC to effectively capture short-chain PFAS, which are considered more challenging for traditional adsorbents.

Moreover, the pH effect on the adsorption performance of AFC-2 was also evaluated in the pH range between 2 and 10, and the bulk ANP was used as the control. As presented in Fig. S29, for ANP, high PFOA removal efficiency was only obtained at pH ≤ 8. As pH increased to 10, the adsorption decreased significantly. Within 30 min, over 95% PFOA was removed by ANP at pH 2, 4, 6, and 8, but the removal ratio dropped to 32% at pH 10. However, for AFC-2, fairly stable PFOA removal efficiency was achieved across the entire pH range, which could also be attributed to the buffering effect of the PVDF membrane, and the protective effect of the PVDF porous shell is further demonstrated here.

### 3.5 Applicability test

We then investigated the regeneration efficiency of spent AFC after the adsorption of PFOA. Five consecutive adsorption/desorption experiments for PFOA were conducted, and methanol was used as a desorption agent. As shown in Fig. S30, the amounts of adsorbed and recovered PFOA were similar over five cycles, and the recovery of PFOA reached nearly 100%, except during the first elution. The slightly lower regeneration efficiency in the first cycle could be attributed to PFOA adsorbed in the innermost part of the AFC channels, which was difficult to desorb in a short time. Furthermore, in all five cycles, AFC-2 reduced the initial concentration of PFOA (1 μg/L) to less than 70 ng/L in 30 min. Based on the above results, the AFC-2 showed robust adsorption performance and could be easily regenerated after adsorption.

To further examine the practical application of AFC, we then used AFC-2 in a continuous-column mode to treat simulated PFAS-polluted water containing PFOA (1 μg/L), HA (5 mg/L), Cl^−^ (5 mg/L), Ca^2+^ (5 mg/L), and HCO_3_^−^ (50 mg/L) (Fig. S31). A commercial granular activated carbon (GAC) column was used for comparison. Results show that the PFOA residue concentration increased sharply in the effluent of the GAC column and remained above 70 ng/L throughout the test (Fig. S32). This might be due to the low binding affinity of GAC for PFAS, which resulted in the failure to effectively capture the substance during the short period of flow through the adsorbent. However, the AFC-2 column generated approximately 18,000 B V of effluent before the breakthrough point occurred. This further demonstrated the advantages of the AFC dual-functional structural design in terms of efficient adsorption of PFAS and resistance to interference. Moreover, a comprehensive comparison was conducted between AFC and other adsorbents regarding cost, selectivity, efficiency, and other aspects. AFC showed superior performance in all aspects (Table S3). Therefore, the AFC-2 demonstrated great stability for the potential application in PFAS treatment.

## 4 Conclusion

**Fig. 6:**
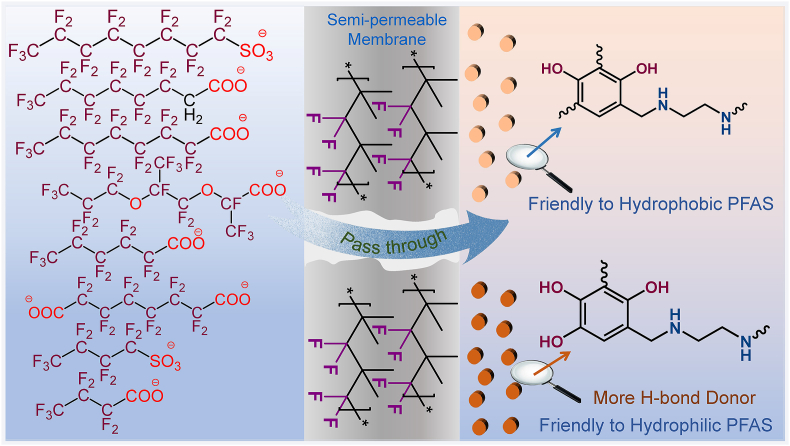
Schematic diagram for the selective adsorption of PFAS by AFC.

In summary, in this study, to simultaneously address theoretical shortcomings and operational challenges in selective PFAS removal, a granular capsule (AFC) with a tunable amine-rich network powder (ANP) core and a porous organic fluorine shell was synthesized. Through systematic experimentation and theoretical validation, the distinct roles of organic fluorine and amine moieties in the adsorption process were thoroughly elucidated, and the interaction mechanism for the selective adsorption of PFAS was proposed at the molecular level. In the composition of AFC, the porous PVDF shell acts as a biomimetic semipermeable membrane, allowing only PFAS to pass through and repelling the coexisting substances due to the fluorophilic effect, thus reducing the interference of the environmental matrix on the adsorption of PFAS by AFC. As the inner core of the AFC, ANP significantly enhances the attraction for the anionic PFAS through hydrogen bonding and van der Waals forces; thus, PFAS can be efficiently captured. Moreover, by enhancing the hydrogen-bonding sites of ANP, the driving force for short-chain PFAS to enter the interior of the capsule is also enhanced, realizing the strong adsorption of a wide range of PFAS (C−F number: 3−9). This study investigates the roles of PVDF and ANP in the selective adsorption of PFAS (Fig. 6). In addition to the theoretical in-depth discussion, several advantages of this novel capsule material are also demonstrated in practical applications. Firstly, the AFC developed in this study maintains the particle morphology of commercial GAC or IX. The granular AFC would not increase the hydraulic retention time during application, which is an important parameter in actual wastewater treatment, and can directly replace GAC or IX currently used in established water treatment infrastructures. Meanwhile, the granular AFC is also conducive to the recycling of particles. Secondly, due to the confined ANP constructed by a porous PVDF shell, AFC can selectively enrich PFAS from complex water matrices. The excellent selectivity for PFAS makes it suitable for pre-treatment or purification of drinking water sources. This study sheds light on the effective treatment of troublesome PFAS chemicals and may stimulate more attention to the materials with confined core-shell structures.

## CRediT authorship contribution statement

**Zhanghao Chen:** Conceptualization, Data curation, Funding acquisition, Investigation, Methodology, Writing – original draft. **Xinhao Wang:** Investigation, Methodology. **Junwen Qi:** Methodology. **Liuqing Huang:** Visualization. **Longgang Chu:** Methodology. **Guixiang Zeng:** Methodology. **Bing Wu:** Supervision, Writing – review & editing. **Juan Gao:** Writing – review & editing. **Jiansheng Li:** Writing – review & editing. **Cheng Gu:** Conceptualization, Funding acquisition, Supervision, Writing – review & editing. **Hongqiang Ren:** Conceptualization.

## Declaration of competing interest

The authors declare that they have no known competing financial interests or personal relationships that could have appeared to influence the work reported in this paper.
