# Supplementary material for: Molecular insights into the selective separation of perfluoroalkyl substances: The construction and application of capsule adsorbents
Source: Eco Environ Health. 2026 Jun 10;5(3):100252. doi: 10.1016/j.eehl.2026.100252 (PMC13380103; doi:10.1016/j.eehl.2026.100252)
Supplement: Multimedia component 1 [file mmc1.docx]

**Supplementary Material for**

**Molecular insights into the selective separation of perfluoroalkyl substances: The construction and application of capsule adsorbents**

Zhanghao Chen^a^, Xinhao Wang^a^, Junwen Qi^b^, Liuqing Huang^a,c^, Longgang Chu^a^, Guixiang Zeng^d^, Bing Wu^a^, Juan Gao^e^, Jiansheng Li^b,^*, Cheng Gu^a,^*, Hongqiang Ren^a^

^a^ State Key Laboratory of Pollution Control and Resource Reuse, School of Environment, Nanjing University, Nanjing 210023, P.R. China

^b^ Key Laboratory of Jiangsu Province for Chemical Pollution Control and Resources Reuse, School of Environmental and Biological Engineering, Nanjing University of Science and Technology, Nanjing 210094, China

^c^ Nanjing Institute of Environmental Sciences, Ministry of Environment and Ecology of China, Nanjing 210042, China

^d^ Kuang Yaming Honors School, Nanjing University, Nanjing 210023, P.R. China

^e^ Key Laboratory of Soil Environment and Pollution Remediation, Institute of Soil Science, Chinese Academy of Sciences, Nanjing 210008, China

*Corresponding author.

**Email:** [lijsh@njust.edu.cn](mailto:lijsh@njust.edu.cn)(J. Li), [chenggu@nju.edu.cn](mailto:xxxxx@xxxx.xxx)(C. Gu)

**Text S1**

*Chemicals:* Perfluorooctanoic acid (PFOA), perfluorooctane sulfonic acid (PFOS), perfluorohexanoic acid (PFHxA), perfluorobutyric acid (PFBA), perfluorobutane sulfonic acid (PFBS), perfluorooctanedioic acid (PFdiCA), hexafluoropropylene oxide trimer acid (HFPO-TA), fluorotelomer carboxylic acid (FTCA (2:6)), formaldehyde, ethylenediamine, resorcinol, oxalic acid and benzoic acid were all obtained from Sigma-Aldrich (Shanghai, China). Polyvinylidene difluoride (PVDF) and commercial granular activated carbon (GAC) were supplied by Macklin Biochemical Co. Ltd. IPA, methanol (MeOH) and acetonitrile (MS grade) were purchased from Merck Inc. (Darmstadt, Germany). Suwannee river fulvic acid (SRFA) and humic acid (SRHA) were supplied by International Humic Substances Society (St. Paul, MN, USA). Hydrochloric acid (HCl, ~37%), sodium hydroxide (NaOH, >98%), sodium chloride (NaCl) and calcium chloride (CaCl_2_) were provided by Nanjing Chemical Reagent Co. All the chemical were used as received without any further purification, and Milli-Q water (18.2 MΩ cm) was used in all experiments.

**Text S2**

*Characterization and Analysis:* The surface morphologies of AFC and FC were characterized by SEM, and the SEM images were collected from a field-emission scanning electron microscope (FEI QUANTA FEG 250, USA). The surface areas and pore structures of AFC and FC were determined by N_2_ adsorption-desorption test based on BJH model at 77 K (NOVA3000, Quantachrome, Boynton Beach, USA). XPS spectra were collected on an X-ray photoelectron spectroscopy (PHI 5000 VersaProbe, Ulvac-PHI). The calibration of the C1s peak was achieved using the adventitious carbon signal at 284.8 eV as a reference point. Furthermore, FTIR spectra were acquired with a Bruker Tensor 27 FTIR spectroscopy (German) in the range of 2200-1000 cm^-1^ with a resolution of 2 cm^-1^, accumulating 32 scans. The procedure for measuring the concentrations of PFAS was described in our previous report [1]. Before measurement, all low-concentration samples were concentrated by solid phase extraction method as reported in the previous literature [2].

**Text S3**

The selective adsorption mechanism of PFOA on AFC was also investigated by *in situ* ATR-FTIR spectroscopy using ANP as adsorbent, and NaCl and FA were utilized as competing substances. As shown in Fig. S21, the increased cumulative peak area of the v(C=O) vibration (1640.9 cm^-1^) represents for the adsorption capacity and the interaction between PFOA and ANP. While, the addition of NaCl and FA significantly inhibits the v(C=O) signal (Fig. S21), which is consistent with the adsorption results, emphasizing the competitive effect of Cl^-^ and FA on PFOA adsorption by ANP. Due to the weak PFOA adsorption on PVDF, *in situ* ATR-FTIR experiments could not directly reflect the signal changes of PFOA on PVDF surface. Both the adsorption of Cl^-^ and FA by PVDF were further studied as shown in Fig. S22, and insignificant adsorption of either NaCl or FA on PVDF was observed, indicating that the co-existing substances mentioned above could not be enriched on PVDF surface. Therefore, the ANP and porous PVDF shell may play different roles for selective removal of PFOA in the AFC composite. In summary, ANP is the critical part for PFOA adsorption, while the porous PVDF shell acts as the shield against the interference from other co-existing substances. The synergistic effect renders AFC the strong adsorption selectivity for PFOA.

**Text S4**

*Theoretical Calculation Methods:* Gaussian 16W program with density functional theory method (M06-2X) was used to investigate the geometry optimizations. Frequency calculations were carried out for each stationary structure to make sure whether it is an equilibrium structure or a transition state. The intrinsic reaction coordination calculations were also performed for each transition state to connect the reactant and product in a specific reaction step. In this study, the M06-2X/6-311+G (d, p) basis set was employed in the calculations. Furthermore, the solvent (H_2_O) effect was considered with the SMD solvation model. Gibbs free energies were evaluated at 298.15 K and 1 atm. To simplify the calculation, PVDF with eight monomers was utilized for Gaussian simulation, and similar operation was reported in a previous study [3]. According to the literature [4], the possible ANP molecular structure was used for simulation as shown below.

Scheme S1. Proposed chemical structures of ANP.

*MD simulations:* All-atom MD simulations were performed to collect the detailed adsorption pathways of PFAS into AFC. Two fixed PVDF chains were used to simulate the PVDF membrane structure, and they were placed in the middle of the box. The left side of the PVDF chains contained six monomolecular phenolic resins, representing the inside of the resin spheres. PFAS and various co-existing ions were placed on the right side of the PVDF chain to represent the external aqueous environment. Water molecules were uniformly placed inside the box and the average density was adjusted close to bulk water at 298.15 K and 1 atm. The simulation box dimensions were 3.0 nm × 6.0 nm × 3.0 nm, and periodic boundary conditions were imposed in all directions. All models were built using the Packmol software package and visual analysis was performed utilizing the VMD software.

MD simulations were conducted by the GROMACS software package. PFAS with different chain lengths and various co-existing ions were placed and frozen at the same specific location to the right-side of PVDF chain, and the distance was changed to allow the PFAS molecule to pass through the PVDF chain and get into the left-side. This process was used to simulate the process of PFAS in real water penetrating the PVDF membrane and entering the interior of the resin sphere. Before MD simulations, all molecules were structurally optimized using Gaussian 16W software. The interactions of the various atoms within the system were carried out using the Amber99sb-ildn force field with the addition of Cl^-^ to neutralize the charges of the system. All simulations were performed at 298.15 K and in NPT system. The system was firstly energy minimized and then equilibrated for 2 ns with a timestep of 2 fs. Finally, the average energy of the system was output and subsequent statistics were performed.

*Weak interaction analysis:* The weak interaction map visualized the region of interatomic interactions through the independent gradient model based on Hirshfeld partition (IGMH) method using the three-dimensional function δ*g*. It represents the interatomic interactions by calculating the overlap of interatomic densities. With the equilibrium structure of the molecule, the greater the overlap of interatomic densities, the higher the degree of interaction. The δ*g* function can be expressed as:

$$\text{δ}\text{g}\left( \text{r} \right)\text{ }\text{= }\text{g}^{\text{IGM}}\left( \text{r} \right) \text{-}\text{ }\text{g}\text{(}\text{r}\text{)}$$

with

$$\text{ }\text{g}\left( r \right) = \left| \sum_{i} \nabla\rho_{i}(\boldsymbol{r}) \right|$$

$$\text{g}^{IGM}\left( \boldsymbol{r} \right) = \sum_{i} \left| \nabla\rho_{i}(\boldsymbol{r}) \right|$$

where *g* represents gradient, which is the sum of the electron density gradients of two atoms, *g^IGM^* is the IGM (independent gradient model) type electron density gradient, r denotes Cartesian coordinate vector; *i* loops over all atoms, and *ρ*_i_ represents the electron density of the *i* atom.

The IGMH method not only shows the interaction region, but also the type and strength of the interactions by projecting the sign(λ_2_)*ρ* function onto the isosurfaces of δg in different colors. λ_2_ is the second largest eigenvalue of the Hessian matrix of electron densities. The λ_2_ with the value less than 0 at a position in the interaction region indicates an attraction between the atoms, while, a value greater than 0 represents a repulsion interaction. *ρ* is the actual electron density of the current system, and a larger value in the interaction region implies stronger interaction. sign(λ_2_)*ρ* function obtained by multiplying sign(λ_2_) and *ρ* distinguishes between the type of interactions and their strengths. The optimized structure used for IGMH analysis was obtained by Gaussian 16W. Structural optimization and frequency calculations were performed at the M06-2X/6-311+G(d,p) level with the addition of the SMD solvation model to account for the effect of water molecules. All IGMH calculations were performed in the Multiwfn software package (version 3.8).

**Text S5**

*PFAS Adsorption assays:* Kinetic and isotherm adsorption experiments were conducted to assess the ACF adsorption performance for PFAS. The bare FC and commercial GAC were also used for comparison. Briefly, the adsorption experiment was conducted in a glass bottle by introducing 10 mg adsorbent into 10 mL solution containing preset concentration of PFAS. The bare FC and commercial GAC were also used as comparison.

*Effect of Solution Chemistry on the Adsorption of PFAS by AFC:* To evaluate the tolerance of AFC based adsorbents for the complexed environmental matrices, the effects from the coexistence of natural organic matter (NOM), inorganic salts, small molecular organic acids (SMOAs) on the system were studied in the presence of 10 mM inorganic salts (NaCl and CaCl_2_), 5 mg L^-1^ NOM (FA and HA) and SMOAs (OA and BA), respectively, and the effect from pH changes was also evaluated by conducting the adsorption experiments in the range of 2 to 10 (2, 4, 6, 8, and 10).

Detailed information for experimental setup and the adsorption capacity calculation are follows: The adsorption experiment was conducted in a 50 mL glass bottle by introducing 30 mg adsorbent into 30 mL solution containing preset concentration of PFAS. The bare FC, ANP and commercial GAC were also used as comparison. For PFOA adsorption kinetics experiments, the initial PFOA concentration was set at 1 μg L^-1^ (2.4 nM), and the solution pH was controlled at 6.0 ± 0.1 by adding HCl/NaOH except special statement. The effects from the coexistence of NOM, inorganic salts and SMOAs on the system were studied in the presence of 10 mM inorganic salts (NaCl and CaCl_2_), 5 mg L^-1^ NOM (FA and HA) and SMOAs (OA and BA), respectively, and the effect from pH changes was also evaluated by conducting the adsorption experiments in the range of 2 to 10. For PFOA isotherm adsorption experiments, the initial concentrations of PFOA ranged from 0 to 800 mg L^-1^, and the dosage of adsorbent was 1 g L^-1^.

*Reusability of AFC:* PFAS-loaded AFC was regenerated by soaking in MeOH for 12 h and recovered by direct filtration. The extracted PFAS were measured by UPLC-MS/MS (PerkinElmer QSlight 210 triple quadrupole mass spectrometer connected with a PerkinElmer Altus A-30 UPLC) to determine the amounts of recovered PFAS, and the PFAS mass balance was calculated to evaluate the desorption efficiency of MeOH for PFAS. After drying, the recovered AFC could be reused as the adsorbent to remove PFAS from water, and the testing procedure was described above.

**Text S6**

In order to gain more insights for PFOA adsorption process in the presence of NaCl and CaCl_2_, the zeta potentials of ANP in bulk water, NaCl and CaCl_2_ solutions were also investigated. As shown in Fig. S20, the zeta potentials of ANP in both NaCl and CaCl_2_ solutions are significantly reduced, which could be explained by the negative ion layer formed by Cl^-^ at the periphery of ANP, thus inducing the repellence of PFOA.

**Text S7**

*Fixed-Bed Column Experiments:* The fixed-bed adsorption experiment was carried out to evaluate the potential of AFC for practical application. The column test was conducted in a glass column as shown in Fig. S31 (120 mm in length and 12 mm in diameter). Each column was packed with 6 mL of solid adsorbent of GCA or AFC-2. The synthetic solution, containing PFOA, HA, NaHCO_3_ and CaCl_2_ was pumped down-flow by a peristaltic pump (LongerPump BT01-100, China) at a constant flow rate of 24 mL h^-1^, and the specific concentrations are indicated in Fig. S31. The empty bed contact time for the column experiment was set as 20 min.


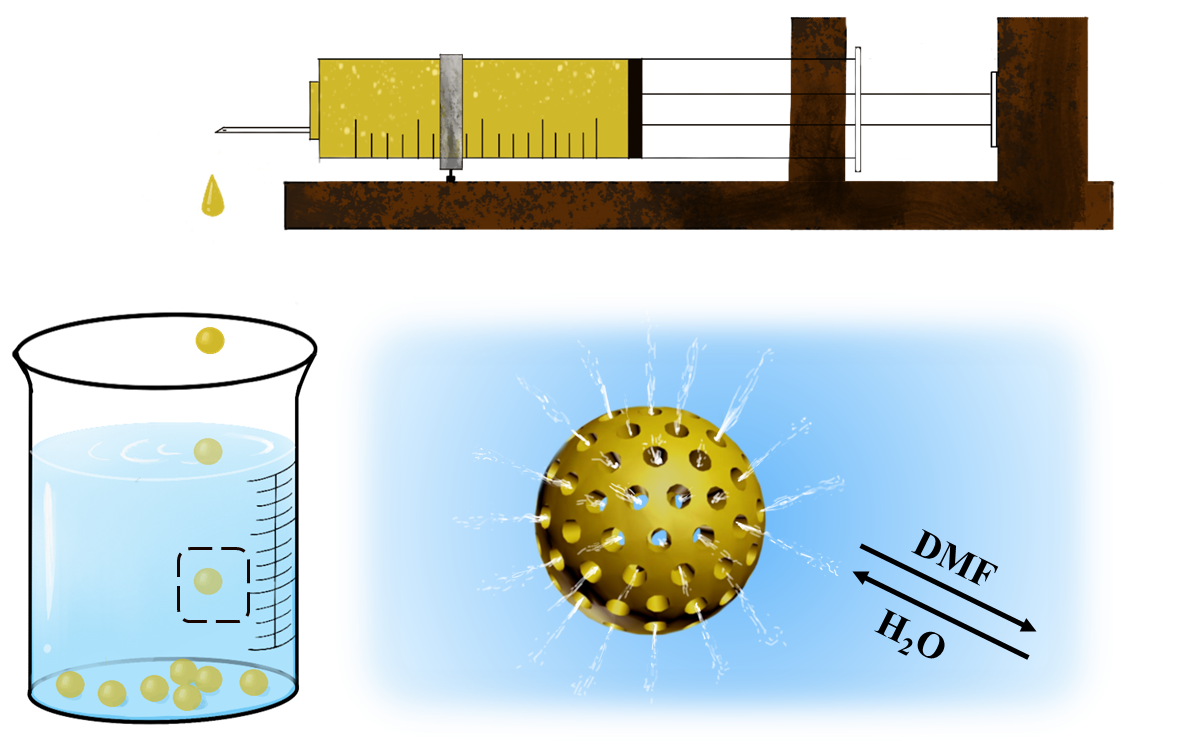


**Fig. S1.** Schematic illustration for the fabrication of AFC.

**
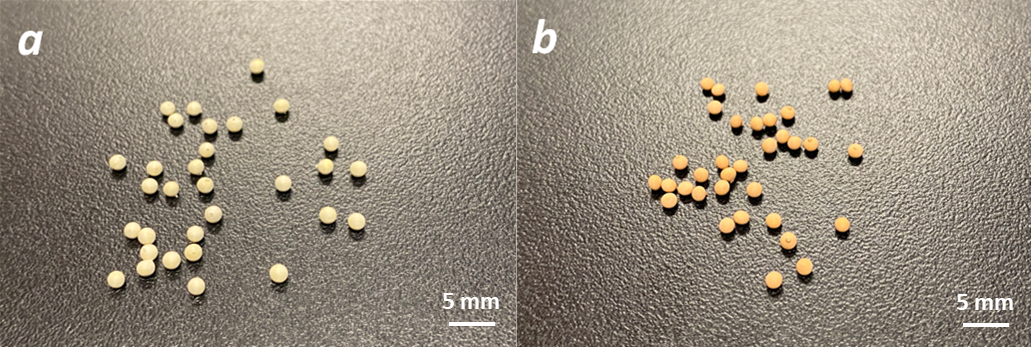
**

**Fig. S2.** The optical images of the synthesized (a) FC and (b) AFC-2.


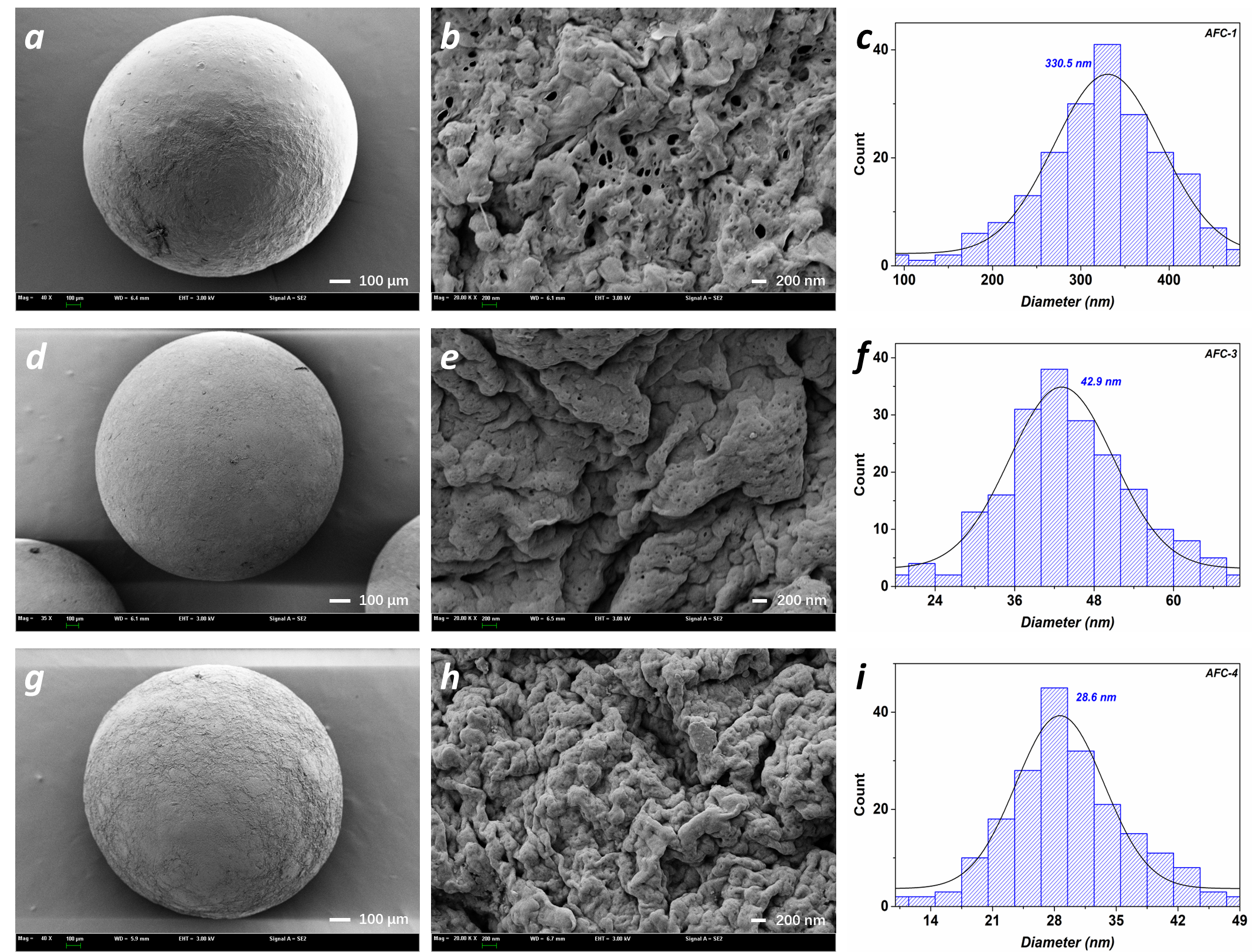


**Fig. S3.** Representative morphologies and surface structures of (a-b) AFC-1, (d-e) AFC-3, and (g-h) AFC-4; and the size distribution histograms of (c) AFC-1, (f) AFC-3, and (i) AFC-4, respectively.


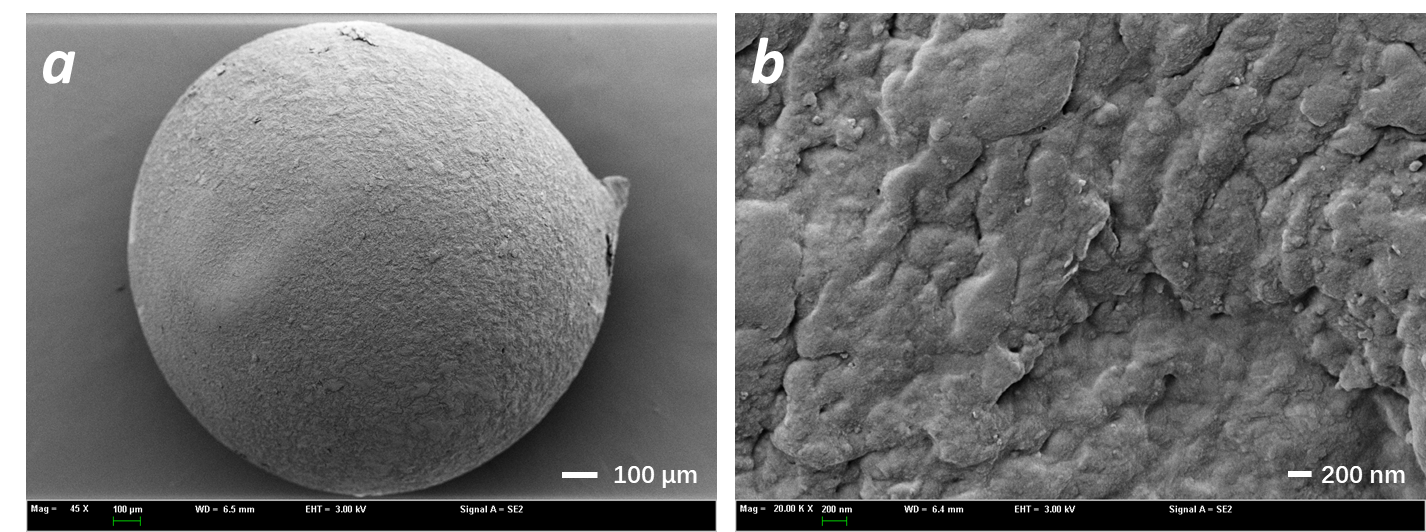


**Fig. S4.** Representative (a) morphology and (b) surface structure of AFC-5.


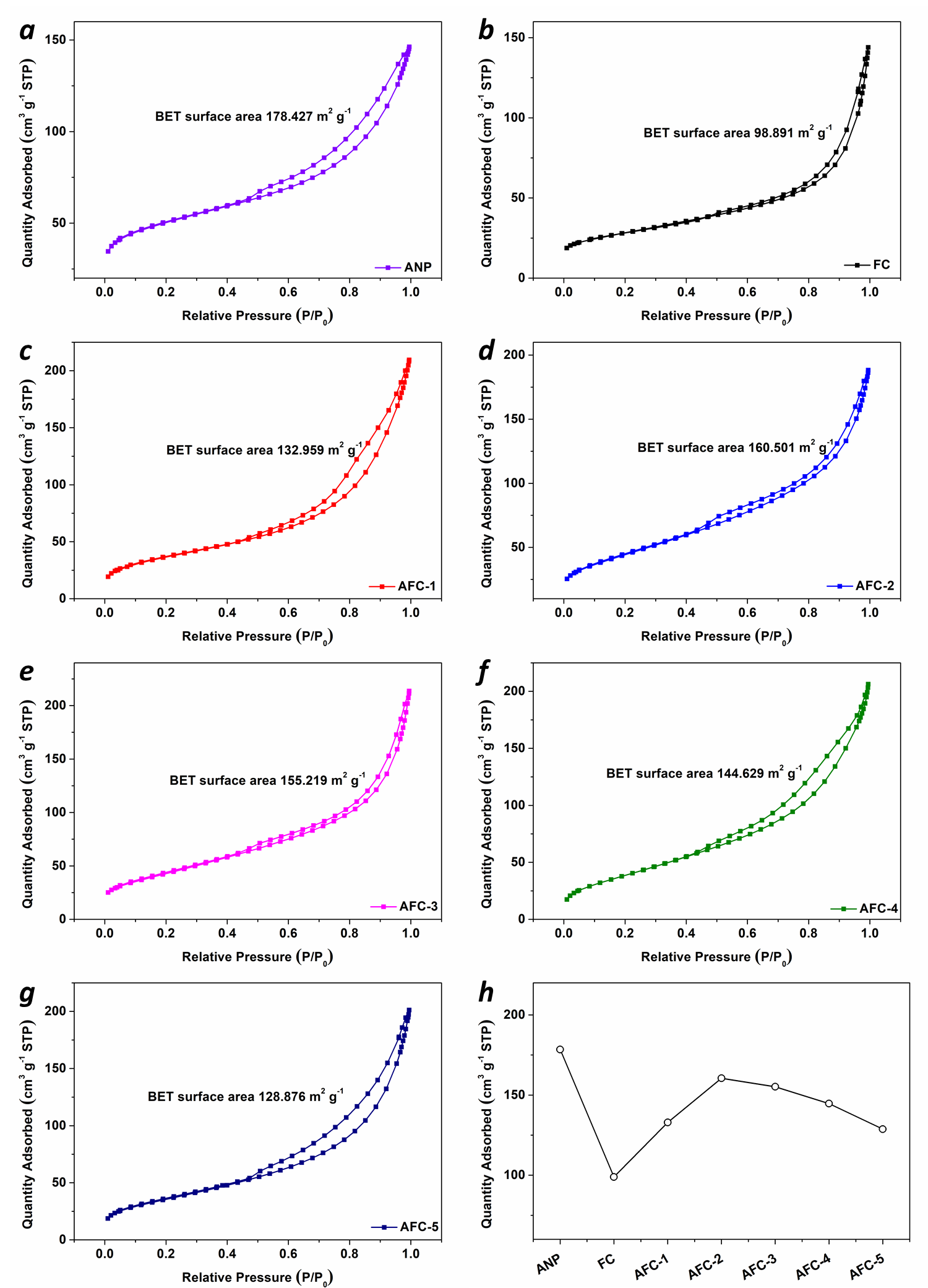


**Fig. S5.** Nitrogen isothermal adsorption-desorption curves of (a) ANP, (b) FC, (c) AFC-1, (d) AFC-2, (e) AFC-3, (f) AFC-4, and (g) AFC-5; (h) the special surface areas of different samples.







**Fig. S6.** XPS survey spectra of (a) bulk FC and AFC-n; (b) the high resolution N1s XPS spectra of bulk FC and AFC-n.





**Fig. S7.** XPS hyperfine spectra of N1s core level (393-406 eV) for ANP.


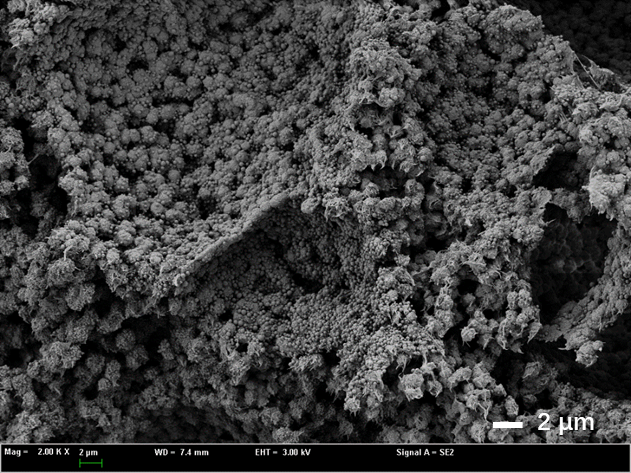


**Fig. S8.** SEM image of AFC-2 cross section.





**Fig. S9.** FTIR spectra of bulk FC and AFC-n.





**Fig. S10.** The surface potentials of bulk ANP and FC vs pH.





**Fig. S11.** Removal of PFOA by ANP, FC, and AFC-n with 30 min of contact time. The initial concentration of PFOA was 1 μg L^-1^, and the dosage of different adsorbents was 1 g L^-1^. The temperature was controlled at 25 ± 1 ℃ on a rotary shaker at 150 rpm, and the solution pH were adjusted to 6 ± 0.1.







**Fig. S12.** ATR-FTIR spectra of PFOA on (a) PVDF film and (b) ANP film. The initial concentration of PFOA was 10 mg L^-1^, and the solution pH was controlled at 6.0 ± 0.1.





**Fig. S13.** Langmuir adsorption isotherms of PFOA by AFC-2 at different temperatures. The initial concentrations of PFOA ranged from 0 to 800 mg L-1. The adsorption experiments of PFOA were conducted at 288, 298 and 308 K, respectively, on a rotary shaker at 150 rpm for 12 h, and the solution pH was adjusted at 6.0 ± 0.1.





**Fig. S14.** The Langmuir adsorption isotherms of PFOA adsorbed by FC and AFC-n. The initial concentrations of PFOA ranged from 10 to 800 mg L^-1^. The adsorption experiment of PFOA was conducted at room temperature (25 ± 1 ℃) on a rotary shaker at 150 rpm for 12 h, and the solution pH was adjusted at 6.0 ± 0.1.

**
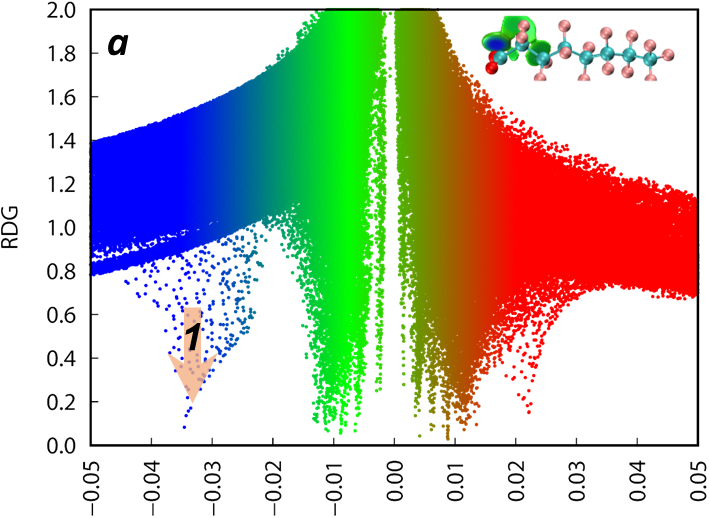
**

**
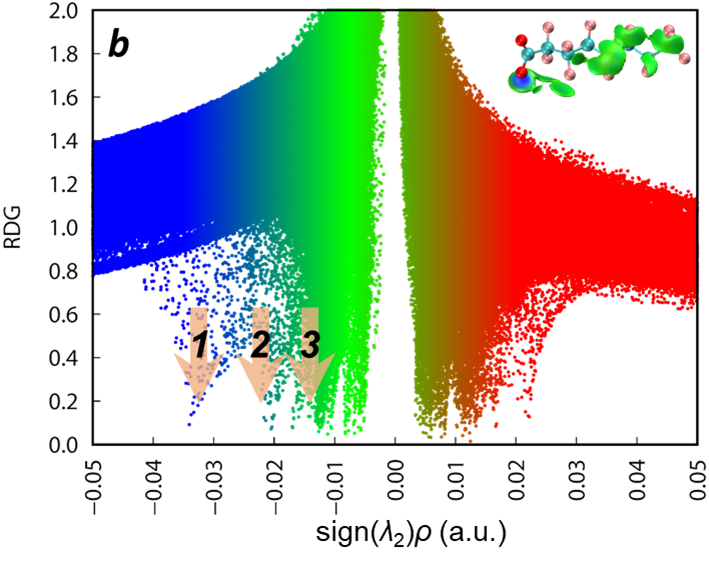
**

**Fig. S15.** The average noncovalent interaction analysis of the interactions between (a) PFOA and single ANP units as well as (b) PFOA and multiple ANP units.





**Fig. S16.** Removal of PFOA as a function of contact time. The initial concentration of PFOA was 1 μg L^-1^, and the dosage of adsorbents (GAC and AFC-2) was 1 g L^-1^. The temperature was controlled at 25 ± 1 ℃ on a rotary shaker at 150 rpm, and the solution pH were adjusted to 6 ± 0.1.





**Fig. S17.** The Langmuir adsorption isotherms of PFOA adsorbed by GAC and AFC-2. The initial concentrations of PFOA ranged from 0 to 800 mg L^-1^. The adsorption experiment of PFOA was conducted at room temperature (25 ± 1 ℃) on a rotary shaker at 150 rpm for 12 h, and the solution pH was adjusted at 6.0 ± 0.1.

**
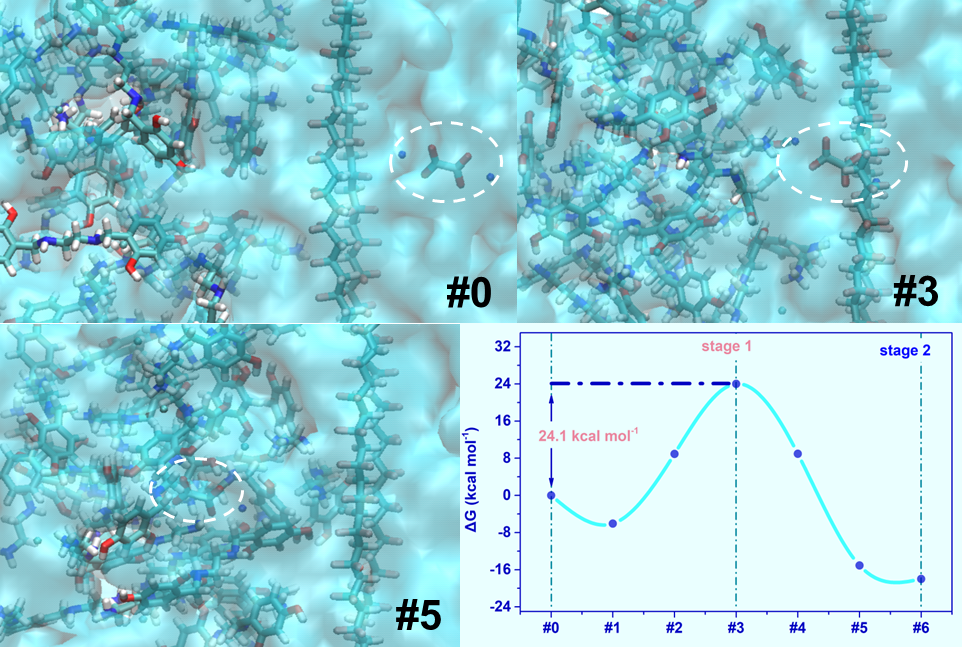
**

**Fig. S18.** Adsorption free energy profiles computed for OA penetration process from bulk solution to AFC inner core.


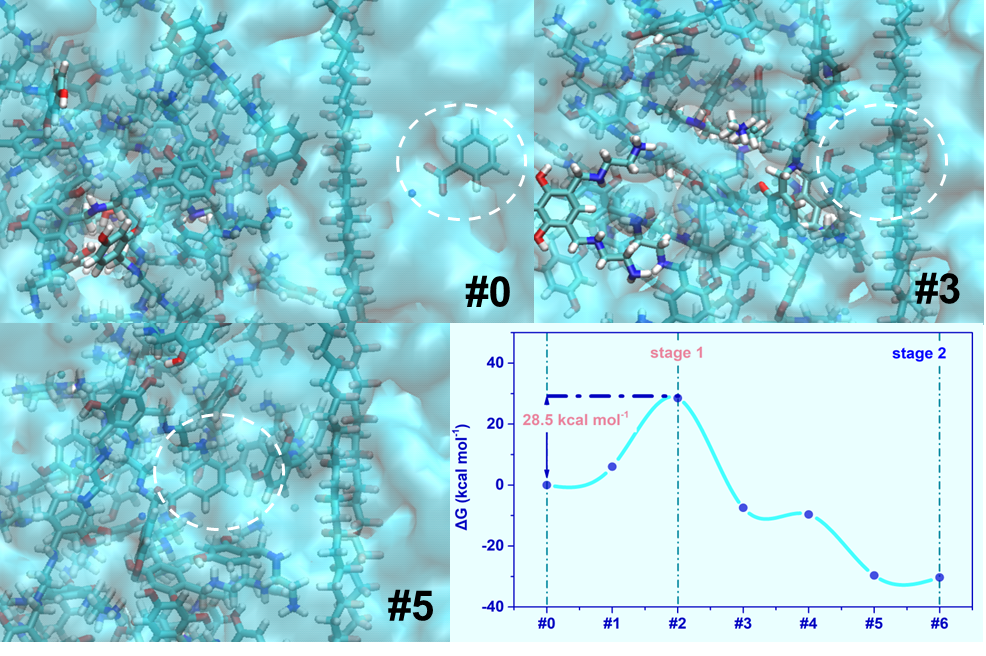


**Fig. S19.** Adsorption free energy profiles computed for BA penetration process from bulk solution to AFC inner core.


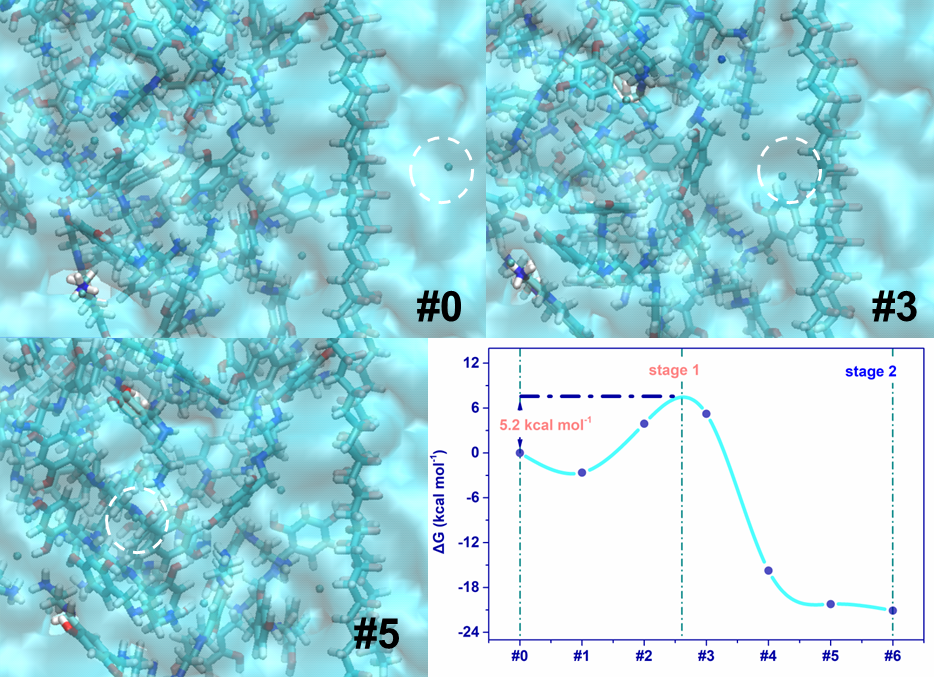


**Fig. S20.** Adsorption free energy profiles computed for Cl^-^ penetration process from bulk solution to AFC inner core.





**Fig. S21.** Surface ζ potentials (mV) of ANP as a function of NaCl and CaCl_2_ concentrations.







**Fig. S22.** ATR-FTIR spectra of PFOA on ANP film in the presence of (a) NaCl and (b) SRFA. The concentrations of PFOA, NaCl and SRFA were 10 mg L^-1^, 10 mM and 5 mg l^-1^, respectively, and the solution pH was controlled at 6.0 ± 0.1.





**Fig. S23.** Adsorption of NaCl and HA by PVDF. Experimental conditions: 1 g L^-1^ PVDF was used as adsorbent, and the initial concentrations of NaCl and HA were 5 mM and 15 mg L^-1^, respectively. The adsorption of NaCl and HA by PVDF was evaluated by measuring the concentration of Cl^-^ and the total organic carbon content before and after adsorption.















**Fig. S24.** PFOA removal by (a) ANP, (b) AFC-1, (c) AFC-2, (d) AFC-3, (e) AFC-4 and (f) AFC-5 with 5 h of contact time. The initial concentration of PFOA was 1 μg L^-1^, and the dosage of adsorbent was 1 g L^-1^; the concentrations of NaCl and CaCl_2_ were 10 mM, and the concentrations of FA, HA, OA and BA were 5 mg L^-1^. The adsorption experiment of PFOA was conducted at room temperature (25 ± 1 ℃) on a rotary shaker at 150 rpm, and the solution pH were controlled at 6.0 ± 0.1.





**Fig. S25.** Various PFAS removal by AFC-2 with 1 h contact time. The initial concentration of all PFAS was 2.4 nM, and the dosage of AFC-2 was 1 g L^-1^. The temperature was controlled at 25 ± 1 ℃ on a rotary shaker at 150 rpm, and the solution pH were controlled at 6.0 ± 0.1.

**
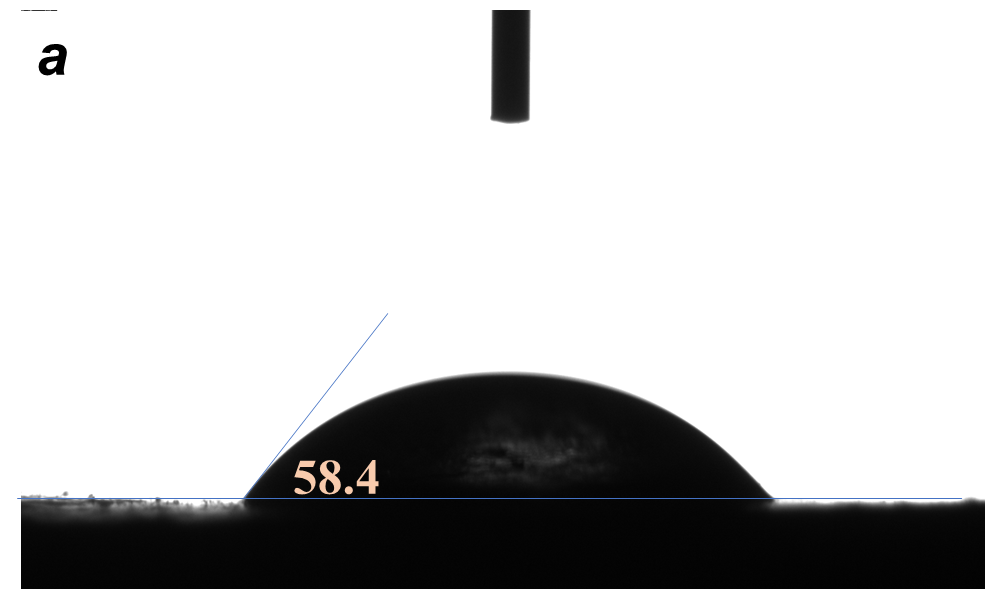

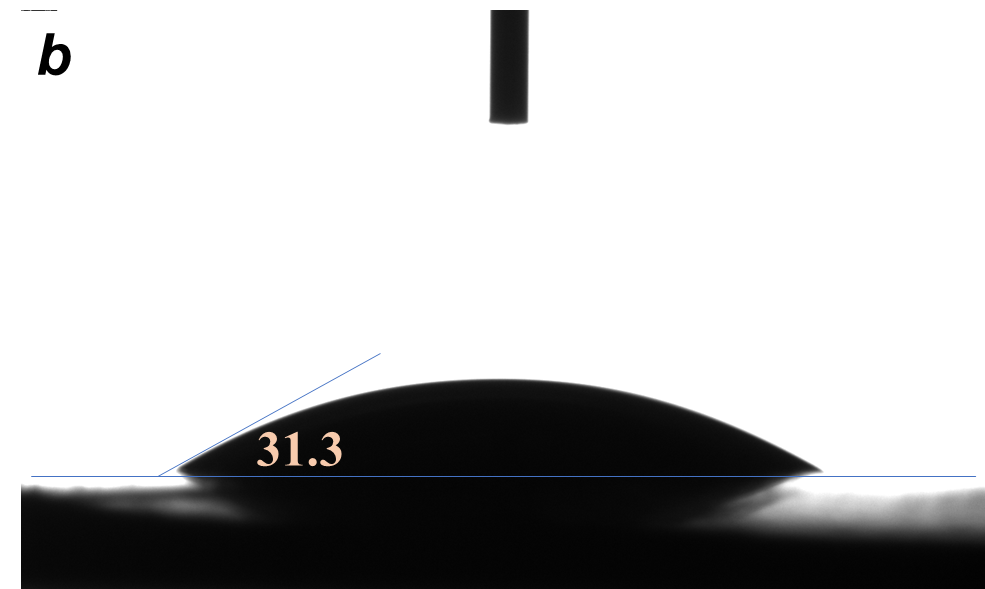
**

**Fig. S26.** Surface hydrophilic and hydrophobic properties of ANP and H-ANP. Digital droplet images of (a) ANP and (b) H-ANP.







**Fig. S27.** (a) Short-chain PFAS removal by AFC-2 and H-AFC with 1 h contact time, and (b) the maximal adsorption capacities for short-chain PFAS on AFC-2 and H-AFC. The initial concentration of all PFAS was 2.4 nM for kinetic adsorption, while from 10 to 800 mg L^-1^ for isotherm adsorption. The dosage of AFC-2 or H-AFC was 1 g L^-1^. The temperature was controlled at 25 ± 1 ℃ on a rotary shaker at 150 rpm, and the solution pH were controlled at 6.0 ± 0.1.

**
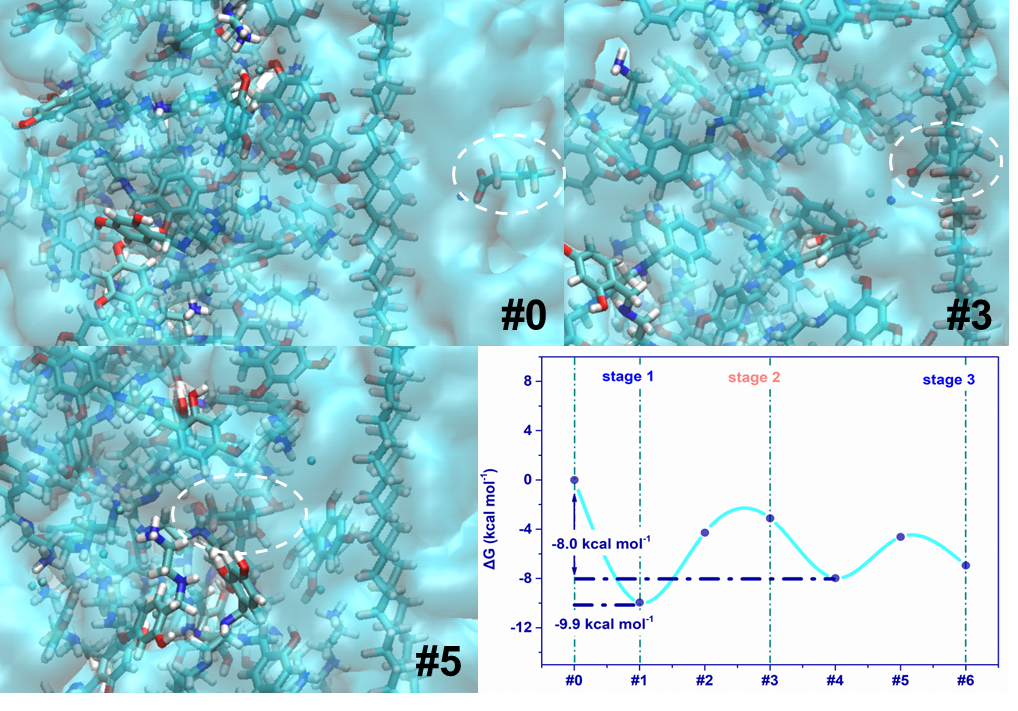
**

**Fig. S28.** Adsorption free energy profiles computed for PFBA penetration process from bulk solution to AFC inner core.





**Fig. S29.** The effect of pH on the removal of PFOA at initial concentration of 1 μg L^-1^. The dosage of AFC-2 was 1 g L^-1^. The adsorption experiment of PFOA was conducted at room temperature (25 ± 1 ℃) on a rotary shaker at 150 rpm, and the solution pH was controlled in the range from 2.0 ± 0.1 to 10.0 ± 0.1.





**Fig. S30.** Regeneration and reuse of AFC-2 by washing with MeOH. The initial concentration of all PFOA in all cycles was 1 μg L^-1^, and the dosage of AFC-2 was 1 g L^-1^. The temperature was controlled at 25 ± 1 ℃ on a rotary shaker at 150 rpm, and the solution pH were adjusted to 6 ± 0.1. During each cycle, AFC-2 was suspended in MeOH to desorb the adsorbed PFOA for 12 h.

**
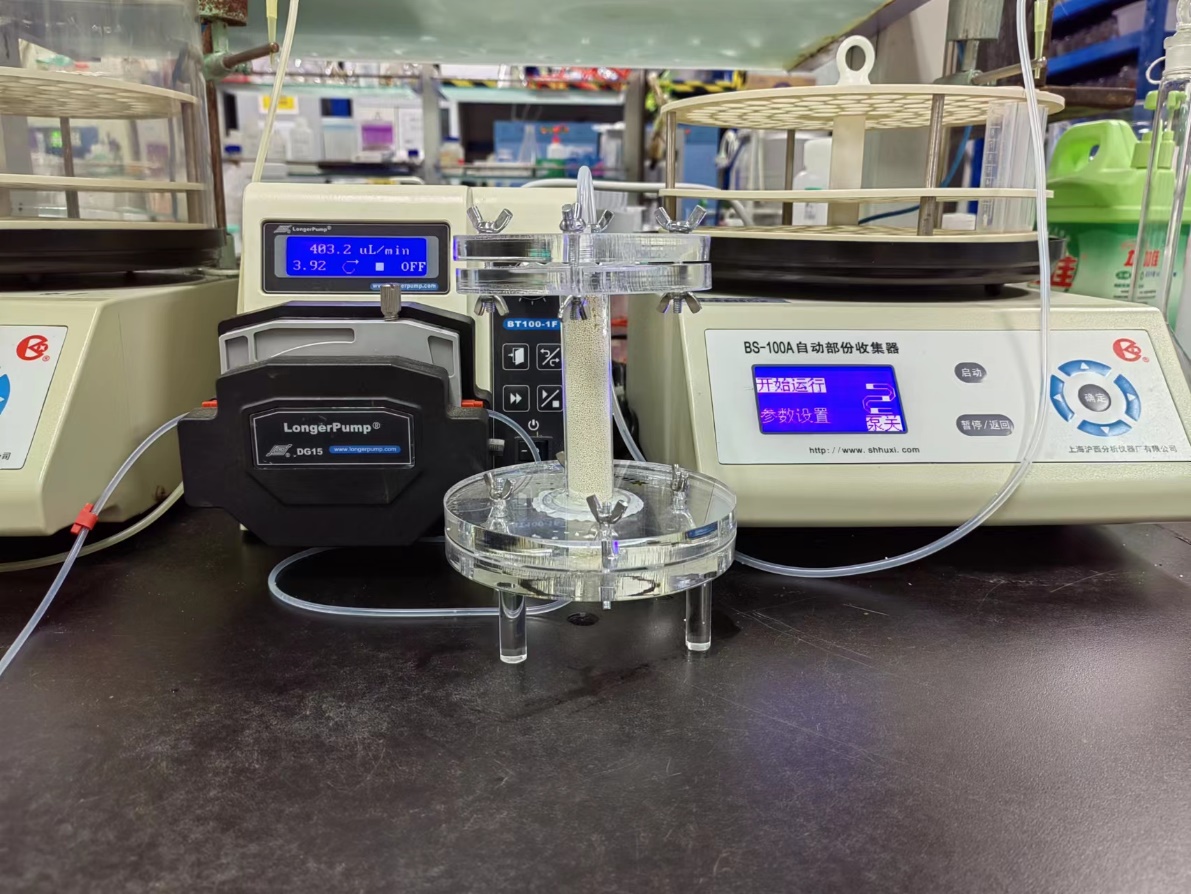
**

**Fig. S31.** The diagram of fixed-bed column test used in the study.





**Fig. S32.** Decontamination of synthetic PFOA-polluted water by AFC-2 and GAC in the column mode. Environmental conditions: the initial PFOA concentration was 1 μg L^-1^, and the solution pH was at 7.1 ± 0.2. The empty bed contact time for the column experiment was set as 20 min.

**Table S1.** Fitted parameters for Langmuir adsorption isotherms of PFOA on FC, AFC-n, GAC and H-AFC.

| adsorbents | PFAS | K_L_ (L mg^-1^) | C_max_ (mg g^-1^) | R^2^ |
| --- | --- | --- | --- | --- |
| FC | PFOA | 0.022 | 114.8 | 0.991 |
| AFC-1 | PFOA | 0.032 | 161.4 | 0.993 |
| AFC-2 | PFOA | 0.037 | 596.2 | 0.996 |
| AFC-3 | PFOA | 0.082 | 557.8 | 0.989 |
| AFC-4 | PFOA | 0.10 | 491.6 | 0.985 |
| AFC-5 | PFOA | 0.096 | 413.1 | 0.968 |
| GAC | PFOA | 0.0036 | 249.7 | 0.984 |
| AFC-2 | PFHxA | 0.0046 | 265.8 | 0.996 |
| AFC-2 | PFBS | 0.0029 | 155.6 | 0.994 |
| AFC-2 | PFBA | 0.0033 | 102.9 | 0.995 |
| AFC-2 | PFdiCA | 0.0013 | 73.7 | 0.993 |
| H-AFC | PFHxA | 0.0086 | 333.0 | 0.996 |
| H-AFC | PFBS | 0.0043 | 235.7 | 0.995 |
| H-AFC | PFBA | 0.0042 | 213.7 | 0.995 |
| H-AFC | PFdiCA | 0.0040 | 199.3 | 0.980 |

The adsorption data were fitted with a Langmuir isotherm model: *Q_e_* = *Q_max_* × *K_L_* × *C_e_* / (1 + *K_L_* × *C_e_*), where, *Q_e_* is the amount of PFOA adsorbed in mg mg^-1^; *Q_max_* is the fitted maximum adsorption amount of PFOA in mg mg^-1^; *K_L_* is the nonlinear adsorption coefficient in L mg^-1^; *C_e_* is the equilibrium concentration in mg L^-1^; and *R^2^* is the coefficient of calculated through the overall regression analysis.

**Table S2.** The structures and chemical characteristics of 8 PFAS used in this study [5,6].

| PFAS | Structure | Chain length | Log K_OW_ |
| --- | --- | --- | --- |
| PFOA |  | 8 | 5.30 |
| PFOS |  | 8 | 6.43 |
| HFPO-TA |  | 9 | 5.55 |
| FTCA |  | 8 | 3.94 |
| PFHxA |  | 6 | 3.71 |
| PFBA |  | 4 | 2.31 |
| PFBS |  | 4 | 2.63 |
| PFdiCA |  | 8 | / |

**Table S3.** Comparison of different adsorbents.

| Adsorbents | Efficiency | Time | Selectivity | Morphology | BET area  (m^2^/g) | Cost | References |
| --- | --- | --- | --- | --- | --- | --- | --- |
| GAC | 20~100% | 5-22 d | No | Particle | ~700 | $1-2/kg | [7] |
| Resin | 65-100% | 1-25 min | No | Particle | / | $5/kg | / |
| MOF | ~100% | ~4 h | Yes | Powder | ~400 | ~$40/kg | [8] |
| COF | ~100% | ~6 h | Yes | Powder | ~1000 | ~$100/kg | [9] |
| Cyclodextrin | ~100% | ~9 h | Yes | Powder | 10-140 | ~$6/kg | [10] |
| AFC-2 | ~100% | 1-15 min | Yes | Particle | 100-160 | $6-8/kg | This study |

**References:**

[1] Chen Z, Zhang S, Wang X, Mi N, Z hang M, Zeng G, et al. Amine-functionalized A-center sphalerite for selective and efficient destruction of perfluorooctanoic scid. Environ Sci Technol 2023;57:10438-10447.

[2] Shi Y, Mu H, You J, Han C, Cheng H, Wang J, et al. Confined water-encapsulated activated carbon for capturing short- chain perfluoroalkyl and polyfluoroalkyl substances from drinking water. Proc Natl Acad Sci 2023;120:e2219179120.

[3] Wang C, Liang S, Bai L, Gu X, Jin X, Xian Z, et al. Structure-dependent surface catalytic degradation of cephalosporin antibiotics on the aged polyvinyl chloride microplastics. Water Res 2021;206:117732.

[4] Rivera A, Ríos-Motta J. An unusual product obtained from condensation between ethylenediamine and formaldehyde in basic medium. Tetrahedron Lett 2005;46:5001-5004.

[5] Pan Y, Zhang H, Cui Q, Sheng N, Yeung LWY, Guo Y, et al. First report on the occurrence and bioaccumulation of hexafluoropropylene oxide trimer acid: An emerging concern. Environ Sci Technol 2017;51:9553-9560.

[6] Feng X, Ye M, Li Y, Zhou J, Sun B, Zhu Y, et al. Potential sources and sediment-pore water partitioning behaviors of emerging per/polyfluoroalkyl substances in the South Yellow Sea. J Hazard Mater 2020;389:122124.

[7] Park M, Wu S, Lopez I, Cahng J, Karanfil T, Snyder S. Adsorption of perfluoroalkyl substances (PFAS) in groundwater by granular activated carbons: Roles of hydrophobicity of PFAS and carbon characteristics. Water Res 2020;170:115364.

[8] Joharian M, Morsali A. Ultrasound-assisted synthesis of two new fluorinated metal-organic frameworks (F-MOFs) with the high surface area to improve the catalytic activity. J Solid State Chem 2019;270:135-146.

[9] Ji W, Xiao L, Ling Y, Ching C, Matsumoto M, Bisbey R, et al. Removal of GenX and Perfluorinated Alkyl Substances from Water by Amine-Functionalized Covalent Organic Frameworks. J Am Chem Soc 2018;140:12677-12681.

[10] Xiao L, Ling Y, Alsbaiee A, Li C, Helbling D. β‑Cyclodextrin Polymer Network Sequesters Perfluorooctanoic Acid at Environmentally Relevant Concentrations. J Am Chem Soc 2017;139:7689-7692.
